# DEVOTE 3: temporal relationships between severe hypoglycaemia, cardiovascular outcomes and mortality

**DOI:** 10.1007/s00125-017-4422-0

**Published:** 2017-09-15

**Authors:** Thomas R. Pieber, Steven P. Marso, Darren K. McGuire, Bernard Zinman, Neil R. Poulter, Scott S. Emerson, Richard E. Pratley, Vincent Woo, Simon Heller, Martin Lange, Kirstine Brown-Frandsen, Alan Moses, Jesper Barner Lekdorf, Lucine Lehmann, Kajsa Kvist, John B. Buse, on behalf of the DEVOTE Study Group

**Affiliations:** 1 Department of Internal Medicine, Medical University of Graz, Auenbruggerplatz 15, A-8036 Graz, Austria; 2 Research Medical Center, Kansas City, MO USA; 3 University of Texas Southwestern Medical Center, Dallas, TX USA; 4 Lunenfeld-Tanenbaum Research Institute, Mount Sinai Hospital, University of Toronto, Toronto, ON Canada; 5 Imperial Clinical Trials Unit, Imperial College London, London, UK; 6 University of Washington, Seattle, WA USA; 7 Florida Hospital Translational Research Institute for Metabolism and Diabetes, Orlando, FL USA; 8 Sanford Burnham Prebys Medical Discovery Institute, Orlando, FL USA; 9 University of Manitoba, Winnipeg, MB Canada; 10 Academic Unit of Diabetes, Endocrinology and Metabolism, University of Sheffield, Sheffield, UK; 11 Novo Nordisk A/S, Søborg, Denmark; 12 University of North Carolina School of Medicine, Chapel Hill, NC USA

**Keywords:** Hypoglycaemia, Insulin therapy, Macrovascular disease

## Abstract

**Aims/hypothesis:**

The double-blind Trial Comparing Cardiovascular Safety of Insulin Degludec vs Insulin Glargine in Patients with Type 2 Diabetes at High Risk of Cardiovascular Events (DEVOTE) assessed the cardiovascular safety of insulin degludec. The incidence and rates of adjudicated severe hypoglycaemia, and all-cause mortality were also determined. This paper reports a secondary analysis investigating associations of severe hypoglycaemia with cardiovascular outcomes and mortality.

**Methods:**

In DEVOTE, patients with type 2 diabetes were randomised to receive either insulin degludec or insulin glargine U100 (100 units/ml) once daily (between dinner and bedtime) in an event-driven, double-blind, treat-to-target cardiovascular outcomes trial. The primary outcome was the first occurrence of an adjudicated major adverse cardiovascular event (MACE; cardiovascular death, non-fatal myocardial infarction or non-fatal stroke). Adjudicated severe hypoglycaemia was the pre-specified secondary outcome. In the present analysis, the associations of severe hypoglycaemia with both MACE and all-cause mortality was evaluated in the pooled trial population using time-to-event analyses, with severe hypoglycaemia as a time-dependent variable and randomised treatment as a fixed factor. An investigation with interaction terms indicated that the effect of severe hypoglycaemia on the risk of MACE and all-cause mortality were the same for both treatment arms, and so the temporal association for severe hypoglycaemia with subsequent MACE and all-cause mortality is reported for the pooled population.

**Results:**

There was a non-significant difference in the risk of MACE for individuals who had vs those who had not experienced severe hypoglycaemia during the trial (HR 1.38, 95% CI 0.96, 1.96; *p* = 0.080) and therefore there was no temporal relationship between severe hypoglycaemia and MACE. There was a significantly higher risk of all-cause mortality for patients who had vs those who had not experienced severe hypoglycaemia during the trial (HR 2.51, 95% CI 1.79, 3.50; *p* < 0.001). There was a higher risk of all-cause mortality 15, 30, 60, 90, 180 and 365 days after experiencing severe hypoglycaemia compared with not experiencing severe hypoglycaemia in the same time interval. The association between severe hypoglycaemia and all-cause mortality was maintained after adjustment for the following baseline characteristics: age, sex, HbA_1c_, BMI, diabetes duration, insulin regimen, hepatic impairment, renal status and cardiovascular risk group.

**Conclusions/interpretation:**

The results from these analyses demonstrate an association between severe hypoglycaemia and all-cause mortality. Furthermore, they indicate that patients who experienced severe hypoglycaemia were particularly at greater risk of death in the short term after the hypoglycaemic episode. These findings indicate that severe hypoglycaemia is associated with higher subsequent mortality; however, they cannot answer the question as to whether severe hypoglycaemia serves as a risk marker for adverse outcomes or whether there is a direct causal effect.

**Trial registration:**

ClinicalTrials.gov NCT01959529

**Electronic supplementary material:**

The online version of this article (10.1007/s00125-017-4422-0) contains peer-reviewed but unedited supplementary material, which is available to authorised users.

## Introduction

People with diabetes are at an increased risk of cardiovascular disease and cardiovascular-related death compared with those without diabetes [1]. Hypoglycaemic events, particularly when severe, have been linked to subsequent adverse cardiovascular outcomes and mortality in individuals with diabetes, although it is currently unknown whether this link is causal, predictive of greater vulnerability or both [2–4].

The Action to Control Cardiovascular Risk in Diabetes (ACCORD) trial was the first large-scale diabetes trial to report that intensive blood glucose management to normalise blood glucose levels (HbA_1c_ target < 6% [42 mmol/mol]) was associated with a significant increase in the risk of cardiovascular-specific mortality, a factor that led to the early termination of the trial [5]. Hypoglycaemia was suggested as a possible mechanism for the increased number of fatal events in the intensive treatment arm of ACCORD, although this association was not clearly demonstrated [6]. However, the ACCORD trial did describe a significantly increased risk of a fatal event after a severe hypoglycaemic event, in both the standard and the intensive treatment arms [6]. Furthermore, a subsequent meta-analysis of several clinical trials and observational studies suggested that severe hypoglycaemia was associated with a higher risk of cardiovascular events [7, 8]. Conclusive evidence of a direct causal relationship between hypoglycaemia and cardiovascular events and mortality is lacking, but experimental evidence in adults without diabetes suggests that hypoglycaemia-induced abnormalities of cardiac repolarisation may contribute to the risk of sudden death [9].

Insulin degludec is a once-daily basal insulin with an ultra-long duration of action, approved for use in adults, adolescents and children with either type 1 or type 2 diabetes [10, 11]. A major clinical benefit of insulin degludec is that it significantly lowers the risk of hypoglycaemia compared with insulin glargine U100 (100 units/ml) [12–15].

The double-blind Trial Comparing Cardiovascular Safety of Insulin Degludec vs Insulin Glargine in Patients with Type 2 Diabetes at High Risk of Cardiovascular Events (DEVOTE) was initiated to assess the cardiovascular safety of insulin degludec compared with insulin glargine U100. DEVOTE demonstrated that in a treat-to-target trial design, insulin degludec was non-inferior to insulin glargine in terms of cardiovascular events and superior with regard to hypoglycaemia risk, with lower rates of both severe and nocturnal severe hypoglycaemia at equivalent glycaemic control [15]. Because of the size and design of the trial and the relatively large number of episodes of severe hypoglycaemia, DEVOTE provides a valuable opportunity to explore the associations of severe hypoglycaemia with cardiovascular outcomes and mortality.

## Methods

The detailed methods of the trial, the trial protocol, the statistical analysis plan and the list of members of the trial teams and committees have been published previously [15, 16]. DEVOTE is registered at ClinicalTrials.gov (number NCT01959529). The trial was conducted in accordance with the Declaration of Helsinki and the ICH Good Clinical Practice Guideline [17, 18]. The protocol was approved by independent ethics committees or institutional review boards for each centre; written informed consent was obtained from each participant before any trial-related activities.

In brief, DEVOTE was a multicentre, prospective, treat-to-target, randomised, double-blind, active comparator cardiovascular outcomes trial, designed to continue until at least 633 major adverse cardiovascular events (MACEs), confirmed by a central, blinded, independent Event Adjudication Committee (EAC), had accrued [15, 16]. All participants had type 2 diabetes treated with at least one oral or injectable glucose-lowering agent with HbA_1c_ ≥ 7.0% (53 mmol/mol), or with ≥ 20 units/day of basal insulin. Patients were eligible for the trial if they either had at least one co-existing cardiovascular or renal condition and were aged ≥ 50 years or had at least one of a list of pre-specified cardiovascular risk factors and were aged ≥ 60 years. Patients were not excluded if they had experienced severe hypoglycaemia prior to randomisation.

Patients with type 2 diabetes at high risk of cardiovascular events were randomised 1:1 to receive either insulin degludec (Novo Nordisk, Bagsværd, Denmark) or insulin glargine (Sanofi, Paris, France), in a blinded fashion, both in identical 100 U/ml, 10 ml vials, administered once daily between dinner and bedtime, in addition to standard care. All patients were allowed to continue their pre-trial glucose-lowering therapy, with the exception of basal and premix insulins, which were discontinued.

The primary adjudicated composite endpoint of DEVOTE was the time from randomisation to the first occurrence of death from cardiovascular causes, non-fatal myocardial infarction or non-fatal stroke. Secondary outcomes included an expanded composite cardiovascular outcome (the primary composite endpoint plus adjudicated unstable angina leading to hospitalisation) and time from randomisation to death from any cause. Adjudication-confirmed severe hypoglycaemia was a pre-specified, multiplicity-adjusted secondary outcome, as defined by the ADA as an episode requiring the assistance of another person to actively administer carbohydrate or glucagon, or to take other corrective actions. Plasma glucose levels may not be available during an event, but neurological recovery after the return of plasma glucose to a normal level is considered sufficient evidence that the event was induced by a low plasma glucose level [19].

In the present analysis, the association between severe hypoglycaemia and either MACE or all-cause mortality was investigated by comparing the risk of an event with or without having experienced severe hypoglycaemia in different time periods (15, 30, 60, 90, 180 and 365 days) prior to the event. Cox regression models were used to analyse these associations for each time period. The indicator of whether a severe hypoglycaemic event had occurred was included in the model as a time-dependent variable. All episodes of severe hypoglycaemia prior to first MACE or all-cause mortality were included in the analysis. Randomised treatment was also included in the model as a fixed factor. For sensitivity analyses, additional baseline information (age, sex, HbA_1c_, BMI, diabetes duration, insulin regimen, hepatic impairment, renal status and cardiovascular risk group inclusion criteria) was accounted for. An investigation with interaction terms indicated that the effect of severe hypoglycaemia on the risk of MACE and all-cause mortality were the same for both treatment arms (insulin degludec and insulin glargine), and so the temporal association for severe hypoglycaemia with subsequent MACE and all-cause mortality is reported for the pooled population. For MACEs occurring on the same day as a severe hypoglycaemic event, 0.5 days was added to the day of the MACE. All analyses were conducted using SAS, version 9.4 (https://www.sas.com/en_ca/software/sas9.html). A *p* value of < 0.05 was considered statistically significant.

## Results

### Overall results from DEVOTE

Detailed results from DEVOTE have been published previously [15]. To summarise, a total of 7637 patients were randomised to either insulin degludec (*n* = 3818) or insulin glargine (*n* = 3819). Of these, 98% completed the final follow-up visit or died during the trial. Vital status was known for 99.9% of participants. The median observation time was 1.99 years in both treatment arms.

The pre-specified analysis demonstrated that insulin degludec was non-inferior to insulin glargine in terms of cardiovascular events (HR 0.91, 95% CI 0.78, 1.06; *p <* 0.001 for non-inferiority), and superior with regard to hypoglycaemia risk, with a lower rate of both severe and nocturnal severe hypoglycaemia (by 40% and 53%, respectively; both *p <* 0.001) [15].

### Severe hypoglycaemia and its association with cardiovascular outcomes and all-cause mortality (secondary analysis)

**Fig. 1:**
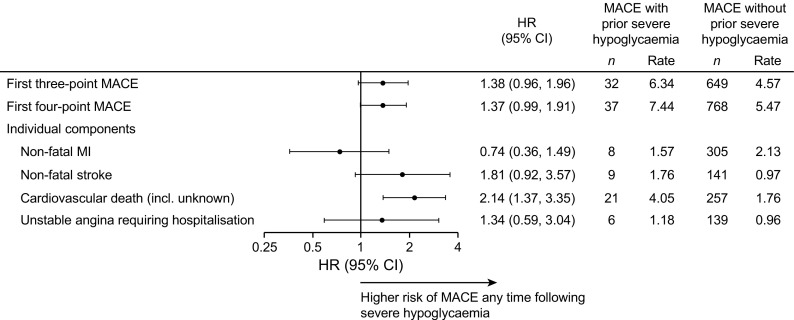
Risk of MACE following a severe hypoglycaemic event. Cardiovascular death includes patients with an unknown cause of death. MI, myocardial infarction; *n*, number of patients; rate, events per 100 patient-years of observation

Of the 681 patients who experienced a MACE and the 439 patients who experienced a severe hypoglycaemic event during the trial, 32 patients had a severe hypoglycaemic event prior to a MACE (14 patients treated with insulin degludec and 18 patients treated with insulin glargine) and 16 patients experienced a MACE prior to severe hypoglycaemia (Table 1 and Electronic supplementary material [ESM] Fig. 1). Of the 423 patients who died from any cause, 38 patients died after experiencing a severe hypoglycaemic event (Table 1). The baseline characteristics of participants who experienced severe hypoglycaemia were not different from those who did not experience severe hypoglycaemia during the trial (ESM Table 1). There was no between-treatment difference in terms of risk of MACE (*p =* 0.679) or all-cause mortality (*p =* 0.209) following severe hypoglycaemia. On this basis, the association between severe hypoglycaemia and time to first MACE or all-cause mortality is reported for the pooled population.

**Table 1:** Overview of outcomes (pooled data)

Outcome	Number of patients	Rate (events/100 PYO)
MACE	681	4.50
Non-fatal myocardial infarction	313	2.07
Non-fatal stroke	150	0.99
Cardiovascular death	278	1.84
Unstable angina requiring hospitalisation	145	0.96
Severe hypoglycaemia	439	4.97
≥ 1 severe hypoglycaemic events prior to MACE	32	6.34
≥ 2 severe hypoglycaemic events prior to MACE	6	4.35
MACE prior to severe hypoglycaemia	16	–
All-cause mortality	423	2.80
Severe hypoglycaemia prior to all-cause mortality	38	7.32

PYO, patient-years of observation

**Fig. 2:**
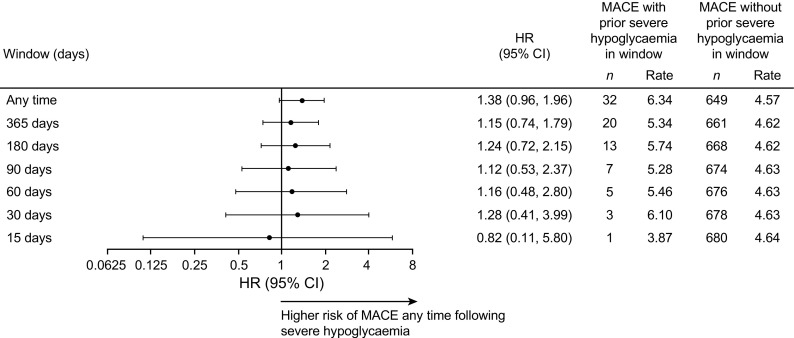
Risk of MACE following a severe hypoglycaemic event by time period. *n*, number of patients; rate, events per 100 patient-years of observation

In the pooled population, there was a non-significant difference in the risk of MACE between participants who had and those who had not experienced severe hypoglycaemia during the trial (HR 1.38, 95% CI 0.96, 1.96; *p =* 0.080) (Fig. 1). A similar result was observed for the expanded four-point MACE definition (HR 1.37, 95% CI 0.99, 1.91; *p =* 0.060) (Fig. 1). When the individual components of the three-point and four-point MACE were investigated, there was a significantly higher risk of cardiovascular death at any time following a severe hypoglycaemic event vs not experiencing a severe hypoglycaemic event (HR 2.14, 95% CI 1.37, 3.35; *p* < 0.001), whereas there was not a significantly higher risk of non-fatal myocardial infarction (HR 0.74, 95% CI 0.36, 1.49; *p* = 0.395), non-fatal stroke (HR 1.81, 95% CI 0.92, 3.57; *p =* 0.085) or unstable angina requiring hospitalisation (HR 1.34, 95% CI 0.59, 3.04, *p =* 0.490) (Fig. 1). When dividing the time period following a severe hypoglycaemic event into time intervals of different durations (15, 30, 60, 90, 180 and 365 days), there was no temporal relationship between severe hypoglycaemia and MACE (Fig. 2). The non-significant relationship between severe hypoglycaemia and MACE was maintained after adjustment for the following baseline characteristics: age, sex, HbA_1c_, BMI, diabetes duration, insulin regimen, hepatic impairment, renal status and cardiovascular risk group inclusion criteria (ESM Table 2). Only a small number of participants experienced a MACE prior to severe hypoglycaemia (*n* = 16) (Table 1), and therefore a possible opposite temporal association could not be analysed. Only three nocturnal severe hypoglycaemic events occurred prior to a MACE and therefore these could also not be analysed separately.

**Fig. 3:**
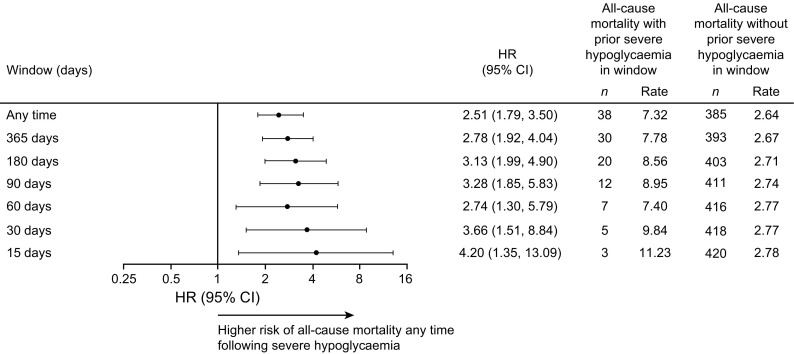
Risk of all-cause death following a severe hypoglycaemic event by time period. *n*, number of patients; rate, events per 100 patient-years of observation

In the pooled population, experiencing severe hypoglycaemia at any time was a significant predictor of all-cause mortality, as the risk for these participants was 2.5-fold that for the participants who did not experience an event (HR 2.51, 95% CI 1.79, 3.50; *p <* 0.001) (Fig. 3). When dividing the time period following severe hypoglycaemia into time intervals of different durations, there was a higher risk of all-cause mortality 15, 30, 60, 90, 180 and 365 days after experiencing severe hypoglycaemia compared with not experiencing severe hypoglycaemia in the same time interval (Fig. 3). The risk appeared to be highest in the shorter-term windows and decreased with the longer-term windows, but remained significant for all (*p* < 0.05 for all). The relationship between severe hypoglycaemia and all-cause mortality was maintained after adjustment for the following baseline characteristics: age, sex, HbA_1c_, BMI, diabetes duration, insulin regimen, hepatic impairment, renal status and cardiovascular risk group inclusion criteria (ESM Table 2). The cause of each death occurring after a severe hypoglycaemic event is listed in ESM Table 3, including the days the severe hypoglycaemic events occurred and the time between the last event and the fatal event. The majority of these fatal events were ascribed to non-cardiovascular reasons (*n* = 17) and the remainder to cardiovascular (*n* = 14) or undetermined causes (*n* = 7).

## Discussion

The results of DEVOTE demonstrated that insulin degludec was superior with regard to severe hypoglycaemia risk at equivalent glycaemic control compared with insulin glargine, thereby confirming observations from earlier studies including the open-label Phase 3a programme and the double-blind, crossover SWITCH trial in patients with type 2 diabetes [12, 13, 15]. The results from the new analyses reported here demonstrate an association between severe hypoglycaemic events and a higher risk of all-cause mortality (*p* < 0.001) in the overall DEVOTE population. In addition, the DEVOTE data suggest that an elevated risk of a fatal event might persist for many weeks and months after a severe hypoglycaemic event, although the highest risk appears to be in the shorter time periods, albeit with small numbers of events. The null hypothesis for the temporal analyses was that there would be an increased risk of an event immediately following severe hypoglycaemia. On this basis we would therefore expect that the ‘any time’ hazard ratio would be lower than those for the shorter time periods, which is what was observed for all-cause mortality. These results are similar to those observed in the Liraglutide Effect and Action in Diabetes: Evaluation of Cardiovascular Outcome Results (LEADER) trial, where a similar temporal analysis was conducted [20].

Hypoglycaemia can have a number of adverse cardiovascular pathological effects in addition to the symptomatic effects experienced by patients. In healthy people, hypoglycaemia triggers a counter-regulatory response with a subsequent increase in catecholamine levels [21–24]. This response induces an increase in myocardial contractility and cardiac output, with a corresponding increase in elasticity of blood vessels and a reduction in central arterial pressure [25–27]. People with a long duration of diabetes may have arterial stiffness or underlying cardiac disease that can compromise the benefits of this counter-regulatory response and lead to adverse outcomes. In an observational study investigating the risk of arrhythmias during spontaneous hypoglycaemia in people with type 2 diabetes at high risk of cardiovascular events, clinically important hypoglycaemia (< 3.5 mmol/l) was common, occurring 6% of the time [28]. These hypoglycaemic events were associated with ECG changes consistent with ischaemia, prolonged QT intervals, repolarisation defects and various cardiac arrhythmias, suggesting that these events could be interconnected. Bradycardia and atrial and ventricular ectopic counts were also significantly higher during episodes of nocturnal vs daytime hypoglycaemia [28]. Furthermore, animal studies have shown that the counter-regulatory sympathoadrenal response is capable of inducing fatal cardiac arrhythmias during severe events [29].

Hypoglycaemia has also been linked to both prothrombotic and proinflammatory effects, which could alter blood flow, increasing the risk of cardiovascular events [21, 30]. The catecholamines released during the counter-regulatory response, as well as the release of coagulation factors and inflammatory cytokines into the circulation, can all increase blood viscosity and promote platelet aggregation and activation, thereby affecting vascular flow [22, 29, 31, 32]. These proinflammatory and procoagulant factors may remain elevated for several days after the hypoglycaemia has resolved, leaving the person vulnerable for some time after the hypoglycaemic event, and potentially contributing to the occurrence of major vascular events [33, 34]. In a clinical setting, analyses from the Veterans Affairs Diabetes Trial (VADT) also demonstrated that a severe hypoglycaemic event was an independent predictor of death at 90 days [19, 35].

There remains considerable controversy over the potential causal relationship between severe hypoglycaemia and adverse cardiovascular events. Several clinical outcome trials and observational studies have shown such an association [23]. In ACCORD, patients who had one or more severe hypoglycaemic episodes had higher rates of death than those who did not experience such episodes [6]. In the Outcome Reduction with Initial Glargine Intervention (ORIGIN) trial, severe hypoglycaemia increased the risk of arrhythmic death (by 77%), all-cause death (by 74%) and cardiovascular death (by 71%) [36]. In addition, a post hoc analysis of the Action in Diabetes and Vascular Disease: PreterAx and Diamicron MR Controlled Evaluation (ADVANCE) trial suggested that severe hypoglycaemia was associated with significantly higher risks for major macrovascular events, cardiovascular death and all-cause death [37]. The major limitation, however, is the inability to definitively attribute the cause of death or cardiovascular event to hypoglycaemia and to determine the exact temporal relationship between hypoglycaemia and a subsequent vascular outcome. A recent analysis of The Examination of Cardiovascular Outcomes with Alogliptin Versus Standard of Care (EXAMINE) trial demonstrated that the relationship between severe hypoglycaemia and MACE was less strong for events occurring after a severe hypoglycaemic event compared with all events and severe hypoglycaemia, suggesting that confounding by comorbidities and hypoglycaemia is important [38]. Indeed, in DEVOTE, the trial population was at particularly high risk of MACE and fatal events, with a long duration of diabetes (> 16 years) and previous insulin use (85%), and the majority of participants with established cardiovascular or chronic kidney disease (85%) [15]. This is in line with other studies that have reported that these patient characteristics were more common amongst those who died following a severe hypoglycaemic event [39]. Similarly, results from a recent post hoc analysis of the LEADER trial data have demonstrated a significantly higher risk of MACE, cardiovascular death and non-cardiovascular death following a severe hypoglycaemic event, particularly in the short term. Those patients who experienced severe hypoglycaemia had a longer duration of diabetes, greater insulin use and a higher incidence of heart failure and kidney disease at baseline [20]. However, confounding by comorbidities does not appear to account for the association alone. A meta-analysis of several clinical trials and observational studies, including over 900,000 patients with type 2 diabetes, observed that severe hypoglycaemia was associated with a higher risk of cardiovascular disease, but that this was unlikely to be entirely a consequence of confounding by comorbid severe illness [7]. In addition, as described above, there is also considerable experimental evidence that hypoglycaemia can lead to arrhythmias and other adverse cardiovascular pathological effects. Overall, it is most likely that hypoglycaemia is just a single contributory factor to cardiovascular events in a much larger multifactorial landscape.

The analyses reported here have several limitations. DEVOTE was designed to collect only severe hypoglycaemic events and therefore the contribution of non-severe events could not be assessed. There are several studies that show that both non-severe and severe hypoglycaemic events are associated with a higher risk of cardiovascular events, hospitalisation and all-cause mortality [40, 41]. Although DEVOTE did not collect non-severe events, the severe events collected were independently adjudicated and provide an accurate view of these events in an at-risk patient population. In addition, while the overall DEVOTE trial population was large, only a small proportion of patients experienced severe hypoglycaemia prior to a MACE or a fatal event, particularly during the shorter time periods (15–60 days), which limits the statistical power of our time-to-event analysis. Furthermore, history of a patient’s previous experience of severe hypoglycaemia prior to the trial was not collected, therefore the contribution of these events to the risk of MACE or all-cause mortality could not be assessed. In addition, it was observed from our analyses and those of other trials that the association between a severe hypoglycaemic event and a higher risk of a fatal event lasts for at least 1 year [20]. It is therefore possible that with a population that mostly used insulin prior to randomisation, confounding from severe hypoglycaemic events prior to trial initiation could have occurred. However, it is also important to note that approximately 30% of the DEVOTE population were on basal insulin alone and 15% were insulin-naive at baseline, and therefore the relative risk of severe hypoglycaemia-induced cardiovascular events in these populations is unlikely to be very high compared with the risk for patients treated with a basal–bolus regimen. Finally, the incorporation of a continuous glucose monitoring element in future trials may also be warranted to provide further information on blood glucose levels at the time of severe hypoglycaemic events as well as up to the time of a MACE.

These analyses also have several strengths. DEVOTE is the first cardiovascular outcomes trial to compare the cardiovascular safety of two basal insulins in a double-blind fashion. Many previous cardiovascular outcome trials have been limited in their potential to explore the relationship between hypoglycaemia and MACE because the therapies used in the two treatment arms were different and hence might have had differing pharmacological influences on the risk of MACE. DEVOTE, a trial comparing two basal insulins, allowed the effect of hypoglycaemia alone to be explored more precisely. Furthermore, the independent adjudication of both severe hypoglycaemic events and MACE provided additional strength to the analyses.

The results from these analyses add to the evidence for an association between severe hypoglycaemia and mortality. However, they do not establish whether hypoglycaemia serves as a risk marker for these events or directly contributes to their occurrence.

## Electronic supplementary material

ESM (PDF 503 kb)

## Abbreviations

ACCORD: Action to Control Cardiovascular Risk in Diabetes
DEVOTE: Trial Comparing Cardiovascular Safety of Insulin Degludec vs Insulin Glargine in Patients with Type 2 Diabetes at High Risk of Cardiovascular Events
LEADER: Liraglutide Effect and Action in Diabetes: Evaluation of Cardiovascular Outcome Results
MACE: Major adverse cardiovascular event
