# Supplementary material for: DEVOTE 3: temporal relationships between severe hypoglycaemia, cardiovascular outcomes and mortality
Source: Diabetologia. 2017 Sep 15;61(1):58–65. doi: 10.1007/s00125-017-4422-0 (PMC6002964; doi:10.1007/s00125-017-4422-0)
Supplement: Supplementary file 1 — (PDF 503 kb) [file 125_2017_4422_MOESM1_ESM.pdf]

DEVOTE 3: temporal relationships between severe hypoglycaemia, cardiovascular outcomes and mortality

Contents

Electronic supplementary material (ESM) Table 1: Baseline characteristics 2

ESM Table 2: Sensitivity analyses adjusting for baseline characteristics 4

ESM Table 3: Listing of all-cause death occurring after a severe hypoglycaemic event...........5

ESM Fig. 1: Time to first Event Adjudication Committee-confirmed major adverse cardiovascular event and prior severe hypoglycaemia by patient...8

Electronic supplementary material (ESM) Table 1: Baseline characteristics

	Patients with severe hypoglycaemic events (*n*=439)	Patients without severe hypoglyaemic events (*n*=7198)
Age (years)	66.1±8.1	64.9±7.3[a]
Men	237 (54.0)	4541 (63.1)
Ethnicity		
Hispanic or Latino	52 (11.8)	1085 (15.1)
Race		
White	334 (76.1)	5441 (75.6)
Black or African American	62 (14.1)	770 (10.7)
Asian	25 (5.7)	751 (10.4)
Other	18 (4.1)	234 (3.3)
Region		
North America	348 (79.3)	4923 (68.4)
Europe	26 (5.9)	849 (11.8)
South America	32 (7.3)	553 (7.7)
India	8 (1.8)	349 (4.8)
Asia excluding India	12 (2.7)	280 (3.9)
Africa	13 (3.0)	244 (3.4)
Diabetes duration (years)	18.6±9.8	16.3±8.8
Smoking status		
Current	54 (12.3)	798 (11.1)
Previous	195 (44.4)	3158 (43.9)
Never	190 (43.3)	3240 (45.0)
Renal status		
Normal renal function (eGFR ≥90 ml^{-1} min^{-1} $(1.73\ m)^{-2}$ per CKD-EPI)	66 (15.3)	1420 (20.0)
Mild renal impairment (eGFR 60–89 ml^{-1} min^{-1} $(1.73\ m)^{-2}$ per CKD-EPI)	152 (35.3)	2966 (41.8)
Moderate renal impairment (eGFR 30–59 ml^{-1} min^{-1} $(1.73\ m)^{-2}$ per CKD-EPI)	192 (44.5)	2512 (35.4)
Severe renal impairment (eGFR <30 ml^{-1} min^{-1} $(1.73\ m)^{-2}$ per CKD-EPI)	21 (4.9)	193 (2.7)
Trial eligibility stratum		
Age ≥50 years and established cardiovascular or chronic kidney disease[b]	390 (88.8)	6119 (85.0)
Age ≥60 years and risk factors for cardiovascular disease[c]	46 (10.5)	1059 (14.7)
Body weight (kg)	95.8±22.9	96.1±22.9
Body mass index (kg/m^2)	34.1±7.1	33.6±6.8
Blood pressure (systolic/diastolic, mmHg)	135.0±18.7/ 74.4±10.4	135.6±18.0/ 76.3±10.4
Pulse (beats/minute)	72.0±10.9	73.2±11.3
HbA_{1c} (%)	8.5±1.7	8.4±1.7

HbA_{1c} (mmol/mol)	69.4±18.6	68.5±18.0
Fasting plasma glucose (mmol/l)	9.4±4.3	9.5±3.9
eGFR (ml^{-1} min^{-1} $(1.73\ m)^{-2}$) based on CKD-EPI	63.1±22.7	68.3±21.4
Total cholesterol (mmol/l)	4.2±1.1	4.3±1.2
Low-density lipoprotein cholesterol (mmol/l)	2.2±0.9	2.2±1.0
High-density lipoprotein cholesterol (mmol/l)	1.2±0.4	1.2±0.3
Triglycerides (mmol/l)	2.0±1.4	2.1±1.8

Full analysis set (all randomised patients); Data listed are number (proportion [%]) or mean ± standard deviation. Percentage refers to the proportion of patients on degludec or glargine treatment

[a]Including 3 patients with age <50 years

[b]Patients with missing age information or age <50 years, but who fulfilled at least one of the inclusion criteria for established cardiovascular/chronic kidney disease were included

[c]Patients with missing age information and who only fulfilled the inclusion criteria for cardiovascular disease risk factors were not included

CKD-EPI, chronic kidney disease epidemiology collaboration formula; eGFR, estimated glomerular filtration rate

ESM Table 2: Sensitivity analyses adjusting for baseline characteristics

	**Hazard ratio (95% CI)**	***p*-value**
**MACE**		
Age	1.01 (1.00, 1.02)	0.0958
Sex	0.82 (0.70, 0.97)	0.0202
HbA_{1c}	1.15 (1.10, 1.20)	<0.0001
Body mass index	1.00 (0.99, 1.02)	0.5204
Diabetes duration	1.00 (0.99, 1.00)	0.3227
Insulin regimen	1.52 (1.19, 1.94)	0.0007
Hepatic impairment	1.99 (1.42, 2.79)	<0.0001
Renal status (eGFR)	0.99 (0.99, 1.00)	0.0004
Cardiovascular risk group inclusion criteria	1.57 (1.19, 2.06)	0.0013
**All-cause mortality**		
Age	1.04 (1.03; 1.06)	<0.0001
Sex	0.71 (0.58, 0.88)	0.0018
HbA_{1c}	1.11 (1.05, 1.18)	0.0003
Body mass index	1.01 (1.00, 1.03)	0.0611
Diabetes duration	1.00 (0.99, 1.01)	0.8854
Insulin regimen	1.33 (0.98, 1.81)	0.0711
Hepatic impairment	3.38 (2.43, 4.72)	<0.0001
Renal status (eGFR)	0.98 (0.98, 0.99)	<0.0001
Cardiovascular risk group inclusion criteria	1.19 (0.86, 1.66)	0.2945

CI, confidence interval; eGFR, estimated glomerular filtration rate; MACE, major adverse cardiovascular event

ESM Table 3: Listing of all-cause death occurring after a severe hypoglycaemic event

Patient	Event	Cause of death	Day of severe hypoglycaemic event	Days from severe hypoglycaemic event to fatal event
1	Non-cardiovascular death	Infection (including sepsis)	321	147
2	Undetermined cause		520<br>519	97<br>98
3	Cardiovascular death	Acute myocardial infarction	53	596
4	Cardiovascular death	Stroke	71	217
5	Non-cardiovascular death	Malignancy	215	156
6	Non-cardiovascular death	Infection (including sepsis)	150	273
7	Non-cardiovascular death	Infection (including sepsis)	658	0
8	Cardiovascular death	Heart failure	303	71
9	Undetermined cause		158	28
10	Cardiovascular death	Heart failure	435	18
11	Non-cardiovascular death	Renal causes	190<br>178<br>170<br>115<br>104	365<br>377<br>385<br>440<br>451
12	Cardiovascular death	Acute myocardial infarction	24	676
13	Cardiovascular death	Sudden cardiac death	246<br>186<br>82	71<br>131<br>235
14	Non-cardiovascular death	Malignancy	604	3
15	Cardiovascular death	Acute myocardial infarction	294	512
16	Undetermined cause		357	298
17	Cardiovascular death	Sudden cardiac death	358<br>307	146<br>197

			139	365
18	Undetermined cause		212	517
19	Non-cardiovascular death	Infection (including sepsis)	368<br>340	78<br>106
20	Non-cardiovascular death	Infection (including sepsis)	407<br>305	360<br>462
21	Undetermined cause		17	573
22	Cardiovascular death	Acute myocardial infarction	7	241
23	Undetermined cause		419<br>316<br>180	282<br>385<br>521
24	Non-cardiovascular death	Malignancy	152	197
25	Cardiovascular death	Sudden cardiac death	27	223
26	Non-cardiovascular death	Infection (including sepsis)	160	580
27	Cardiovascular death	Sudden cardiac death	822	33
28	Non-cardiovascular death	Other non-cardiovascular	288	148
29	Cardiovascular death	Sudden cardiac death	412	161
30	Non-cardiovascular death	Pulmonary causes	753<br>426	31<br>358
31	Cardiovascular death	Heart failure	326	107
32	Non-cardiovascular death	Infection (including sepsis)	15	92
33	Non-cardiovascular death	Malignancy	454<br>85	79<br>448
34	Non-cardiovascular death	Other non-cardiovascular	525<br>522	10<br>13
35	Undetermined cause		20	75
36	Non-cardiovascular death	Infection (including sepsis)	290<br>289	190<br>191

37	Non-cardiovascular death	Pulmonary causes	46<br>38	398<br>406
38	Cardiovascular death	Sudden cardiac death	86	402

Cause of death determined by the Event Adjudication Committee

ESM Fig. 1: Time to first Event Adjudication Committee-confirmed major adverse cardiovascular event and prior severe hypoglycaemia by patient

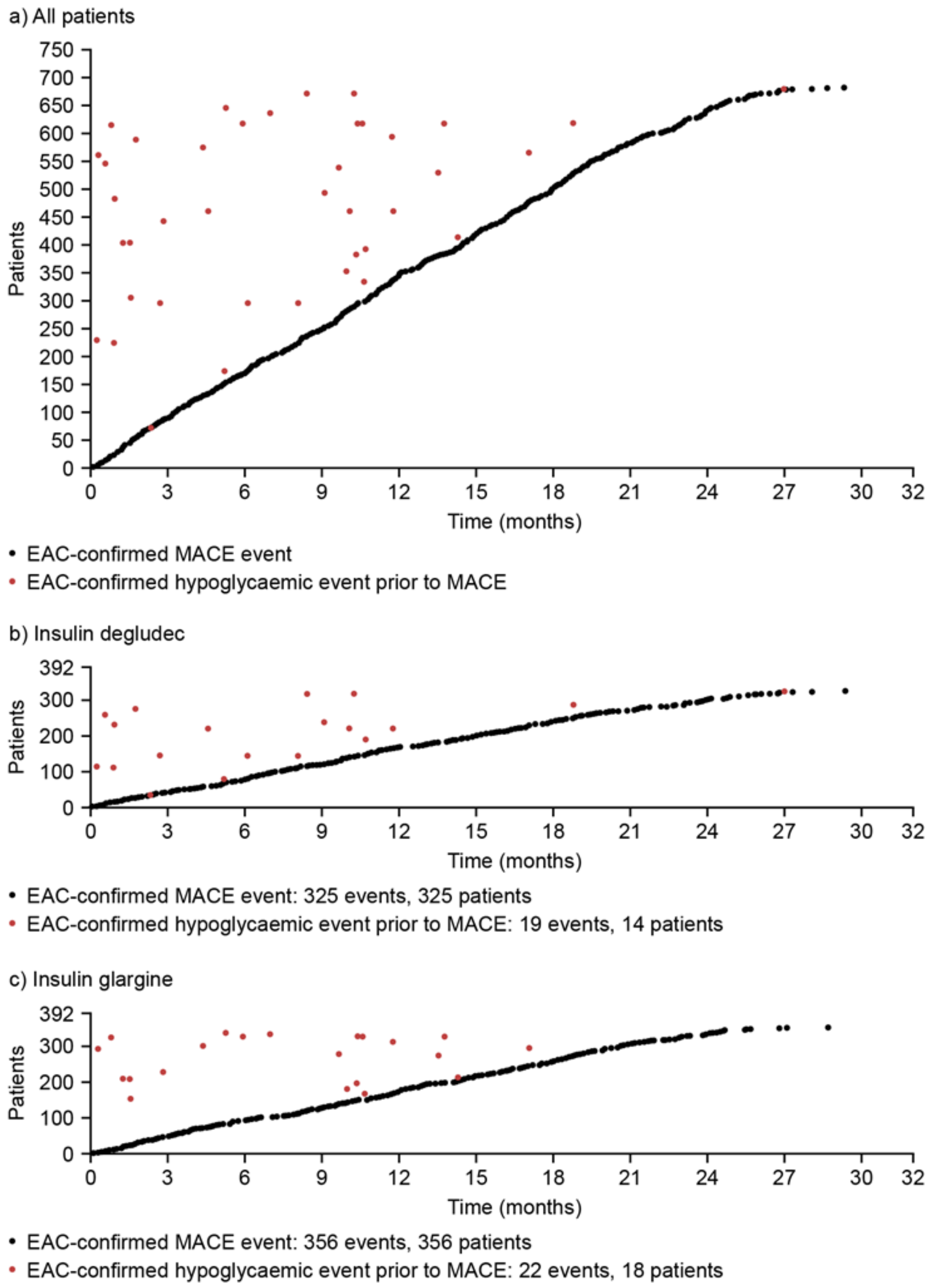

Events for all patients are shown in panel (a) and are split by treatment arm in panels (b) and (c).
EAC, Event Adjudication Committee; MACE, major adverse cardiovascular event
